# Insulin Receptor Substrate 1 Is Involved in the Phycocyanin-Mediated Antineoplastic Function of Non-Small Cell Lung Cancer Cells

**DOI:** 10.3390/molecules26164711

**Published:** 2021-08-04

**Authors:** Shuai Hao, Qiancheng Li, Yuanpu Liu, Fannian Li, Qi Yang, Jing Wang, Chengtao Wang

**Affiliations:** Beijing Advanced Innovation Center for Food Nutrition and Human Health, Beijing Engineering and Technology Research Center of Food Additives, Beijing Technology and Business University, Beijing 100048, China; haoshuai@btbu.edu.cn (S.H.); liqcheng686@163.com (Q.L.); lyppure@163.com (Y.L.); lifannian@163.com (F.L.); yangqi7172021@163.com (Q.Y.); trotwj960@163.com (J.W.)

**Keywords:** phycocyanin, non-small cell lung cancer (NSCLC), proliferation, migration, insulin receptor substrate 1 (IRS-1), antineoplastic

## Abstract

Phycocyanin, derived from marine algae, is known to have noteworthy antineoplastic properties. However, the underlying mechanism involved in phycocyanin-mediated anti-growth function on non-small cell lung cancer (NSCLC) cells is still ambiguous. Here, we investigated the mechanism of action of phycocyanin on H1299, A549, and LTEP-a2 cells. According to the results obtained, insulin receptor substrate 1 (IRS-1) expression was reduced by phycocyanin. Cell phenotype tests showed that siRNA knockdown of IRS-1 expression significantly inhibited the growth, migration, colony formation, but promoted the apoptosis of NSCLC cells. Meanwhile, phycocyanin and IRS-1 siRNA treatment both reduced the PI3K-AKT activities in NSCLC cells. Moreover, overexpression of IRS-1 accelerated the proliferation, colony formation, and migration rate of H1299, A549, and LTEP-a2 cells, which was contradicting to the knockdown results. Overall, this study uncovered a regulatory mechanism by which phycocyanin inhibited the growth of NSCLC cells via IRS-1/AKT pathway, laying the foundation for the potential target treatment of NSCLC.

## 1. Introduction

The incidence and mortality rate of lung cancer is increasing at an alarming rate worldwide [1]. Worldwide, an estimated 19.3 million new cancer cases and almost 10.0 million cancer deaths occurred in 2020 [2]. Lung cancer is the leading cause of cancer death (18%) and the second commonly diagnosed cancer (11.4%) in the past year [2,3,4]. Similar to most malignant tumors, lung cancer is composed of cell subpopulations with different molecular characteristics, leading to heterogeneity within the tumor [5]. Non-small cell lung cancer (NSCLC), one of the subtypes of lung cancer, takes up approximately 85% of lung cancer cases [6], with characteristics of such as metastasis, low cure rate, and high mortality [7]. In recent years, substantial progresses have been made in the emergence of various targeted therapies and the effective application of immunotherapy on NSCLC [8]. However, the main challenges still remain, such as how to identify new driver genes to expand the population benefiting from targeted therapy, and how to search for better predictors or novel functional factors for NSCLC therapies [9]. Therefore, continuous drug development provides increasing patients with the hope of prolonging survival.

The insulin receptor substrate (IRS) family is cytoplasmic adaptor proteins, which play important roles as downstream signaling intermediates of cell surface receptors, and many of which have been reported related to cancers [10]. The IRS protein cascades are the most valuable protein cascades in the insulin signaling pathway, including IRS-1, IRS-2, IRS-3, IRS-4, IRS-5, and IRS-6 [11], of which, IRS-1 and IRS-4 are most associated with tumor growth and proliferation, and IRS-2 is the most frequently associated with tumor movement and invasion [12]. IRS-1, one of the cytoplasmic adaptor proteins in IRS family, is found in insulin-stimulated Fao liver cancer cells for the first time [10], which was derived from insulin-stimulated rat liver [13] and 3T3-L1 cells [14]. It is reported that the expression of IRS-1 is increased in hepatocellular [15], pancreatic [16], and prostate cancer [17], while its expression is negatively correlated with tumor progression in breast [18] cancer. The specific expression and function of IRS-1 in human cancer has aroused our continuous attention to follow-up trials.

Phycocyanin, a type of natural blue pigment, mainly exists in Spirulina and is used as dietary supplement in many countries [19]. Recently, phycocyanin has attracted great attention due to its non-toxic and functional characteristics. The therapeutic value of phycocyanin has been widely confirmed [20] including antioxidant [21], anti-inflammatory [22], anticancer [23], hypolipidemic [24], neuroprotective [25], and immunomodulatory effects [26]. In particular, phycocyanin was proved to exert anti-cancer effect on a variety of cancer types such as breast cancer [27], liver cancer [28], NSCLC [29], and colon cancer [30] in vivo and in vitro. Strikingly, although the antineoplastic function of phycocyanin on NSCLC has been widely studied, the study mainly focused on the phenotype analysis. The underlying inhibitory mechanism of phycocyanin in NSCLC is still required further investigation. In this work, the anticancer mechanism of phycocyanin in NSCLC was analyzed based on H1299, A549, and LTEP-a-2 NSCLC cells. The results were expected to lay a theoretical foundation for the future treatment of NSCLC and the utilization of phycocyanin.

## 2. Results

### 2.1. IRS-1 Expression Is Downregulated by Phycocyanin in NSCLC

**Figure 1:**
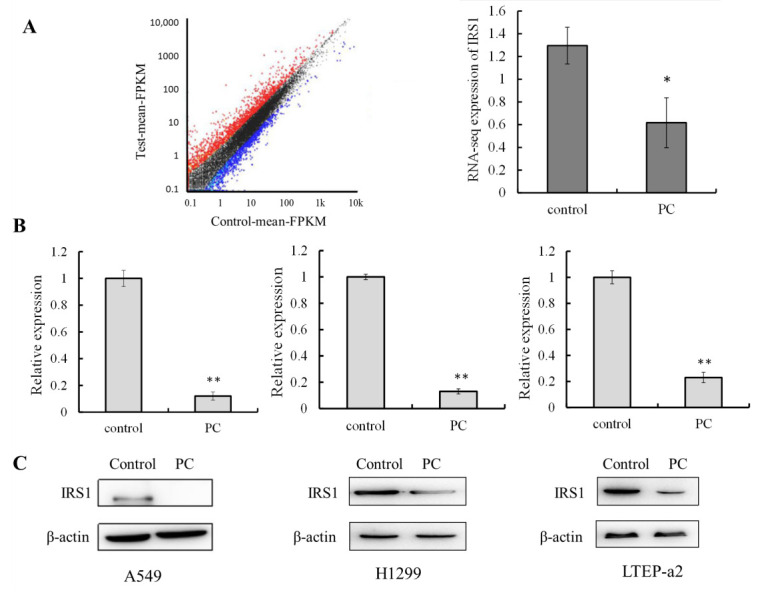
IRS-1 expression is downregulated by phycocyanin in NSCLC. (**A**) mRNA-seq results of IRS-1 expression in A549 cells after 4.8 μM phycocyanin treatment. (**B**,**C**) qRT-PCR and Western blot analysis of IRS-1 expressions after 4.8 μM phycocyanin treatment in A549, H1299, and LTEP-a2 cells. PC, phycocyanin. * *p* < 0.05; ** *p* < 0.01.

Previously, we have demonstrated that phycocyanin (4.8 μM) exerted significantly antineoplastic function on multiple NSCLC cell lines including A549 (Figure S1), H1299, and LTEP-a2 cells [31]. A RNA-seq method was performed to further explore the mechanism of phycocyanin on A549 cell line. A strict comparison at adjusted *p* ≤ 0.05 and log2FC fold change ≥1 (for upregulation) or ≤−1 (for downregulation), was made to determine the differentially expressed genes (DEGs) between phycocyanin-treated (4.8 μM) and untreated groups. The list of DEGs is presented in Supplementary Table S1. In total, 2970 genes are significantly differentially expressed, including 1431 downregulated and 1539 upregulated genes. IRS-1 (marked in yellow, line 888 in Table S1), one of the differentially expressed genes, is significantly downregulated (0.61 ± 0.21) compared with control group (1.32 ± 0.15, Figure 1A). To verify whether phycocyanin could reduce the expression of IRS-1 in NSCLC cells, we performed qRT-PCR and Western blot assay. As shown in Figure 1B,C, the expressions of IRS-1 significantly decreased after phycocyanin treatment in A549, H1299, and LTEP-a2 cells, which was consistent with transcriptome results. These results suggested that IRS-1 might participate in the antineoplastic function of phycocyanin in NSCLC cells.

### 2.2. Knockdown of IRS-1 Expression Promotes Apoptosis of NSCLC Cells

**Figure 2:**
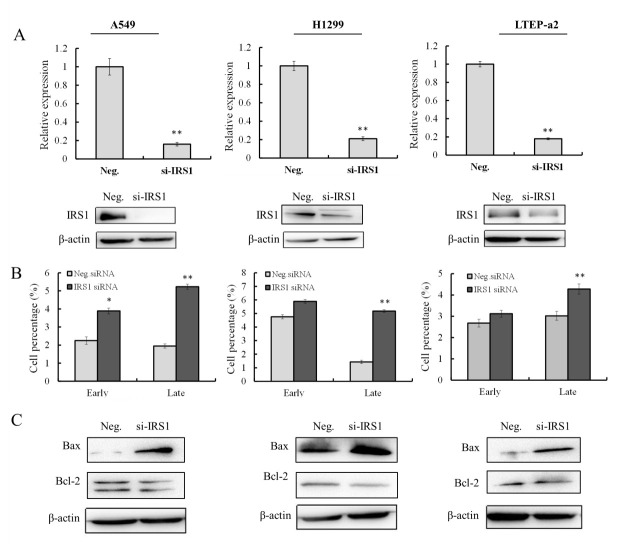
Knockdown of IRS-1 expression promotes apoptosis of NSCLC cells. (**A**) The silencing efficacy detection of IRS-1 transcription (24 h) and protein (48 h) levels after 50 nM siRNA transfection in A549, H1299, and LTEP-a2 cells. (**B**) Apoptosis analysis of A549, H1299, and LTEP-a2 cells after 50 nM siRNA knockdown of IRS-1 expressions for 48 h. (**C**) Western blot analysis of Bax and Bcl-2 expressions after 50 nM IRS-1 siRNA transfection in A549, H1299 and LTEP-a2 cells for 48 h. * *p* < 0.05; ** *p* < 0.01.

The functions of the ectopic expression of IRS-1 on cell phenotype were explored using siRNA transfection. The silencing efficacy of IRS-1 was validated by qRT-PCR and Western blot analysis (Figure 2A). As a result, compared with control groups, the expressions of IRS-1 were significantly decreased after siRNA transfection in A549, H1299, and LTEP-a2 cells, indicating that IRS-1 expression was successfully silenced. As shown in Figure 2B, siRNA knockdown of IRS-1 expression significantly promoted the proportion of late apoptotic A549 (5.26% ± 0.37%), H1299 (5.24% ± 0.12%), and LTEP-a2 (4.18% ± 0.04%) cells, respectively. In addition, the proportion of early apoptotic A549 cells was also significantly induced (3.72% ± 0.14%) after IRS-1 silencing. It is worth noting that although the late apoptotic rates are increased in RRS-1 knockdown cells, they are far less than that in phycocyanin-treated H1299 (11.30% ± 0.16%) and LTEP-a2 (14.50% ± 0.68%) cells. This indicates that phycocyaninexerts antineoplastic effects of NSCLC cells via complex mechanisms, and IRS-1 might be just one of the potential regulatory factors of phycocyanin. Subsequently, the expressions of apoptosis markers were analyzed at protein levels (Figure 2C). It was shown that siRNA knockdown of IRS-1 could significantly promote the expressions of pro-apoptotic gene Bax, whereas slightly reduce the expressions of anti-apoptotic gene Bcl-2. These results indicated that the ectopic expression of IRS-1 affected the apoptosis of A549, H1299, and LTEP-a2 cells, which might be one of the potential mechanisms of phycocyanin on NSCLC.

### 2.3. Knockdown of IRS-1 Expression Inhibits the Proliferation, Migration, and AKT Activity of NSCLC Cells

**Figure 3:**
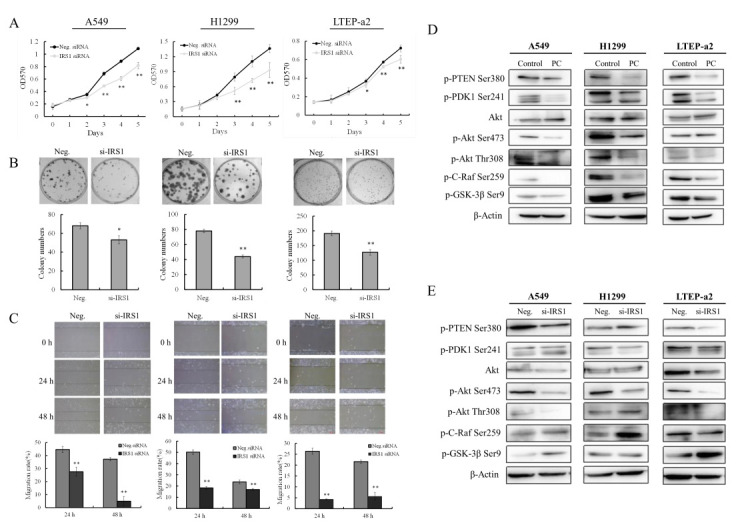
Knockdown of IRS-1 expression inhibits the proliferation, migration and AKT activity of NSCLC cells. (**A**) Proliferation assay of NSCLC cells after 50 nM IRS-1 siRNA treatment. (**B**) Colony formation assay of NSCLC cells after 50 nM IRS-1 siRNA treatment. (**C**) Wound-healing assay of NSCLC cells after 50 nM IRS-1 siRNA treatment. (**D**,**E**) Western blot assay of PI3K-AKT signaling proteins of NSCLC after 4.8 μM phycocyanin and 50 nM IRS-1 siRNA treatment for 48 h, respectively. PC, phycocyanin. * *p* < 0.05; ** *p* < 0.01.

The cell growth curve showed that siRNA knockdown of IRS-1 expression significantly inhibited the proliferation abilities of A549, H1299, and LTEP-a2 cells in vitro (Figure 3A). Besides, the colony forming abilities of NSCLC cells were also suppressed after IRS-1 siRNA treatment (Figure 3B), suggesting that IRS-1 influences the unanchored growth ability of NSCLC cells. The wound-healing assay showed that silencing IRS-1 expression significantly reduced the migration abilities of NSCLC cells at 24 and 48 h, respectively (Figure 3C). The PI3K-AKT signaling was involved in multiple regulatory processes including cell proliferation, migration and apoptosis [32]. To explore whether phycocyanin and IRS-1 regulate PI3K-AKT pathway in NSCLC cells, we further analyzed the phosphorylation levels of related proteins. As shown in Figure 3D,E, phycocyanin and IRS-1 siRNA treatment both reduced the phosphorylation levels of AKT proteins in A549, H1299, and LTEP-a2 cells, indicating phycocyanin might inhibit proliferation and migration of NSCLC cells through downregulating IRS-1/AKT activities.

### 2.4. Overexpression of IRS-1 Promotes Proliferation and Migration of NSCLC Cells

**Figure 4:**
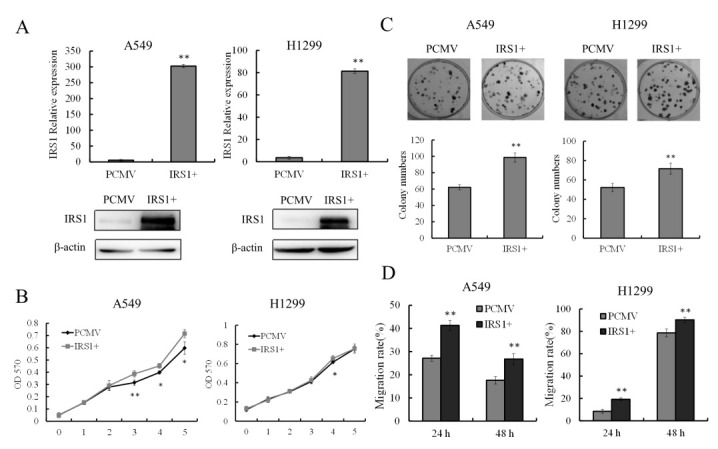
Overexpression of IRS-1 promotes proliferation and migration of NSCLC cells. (**A**) Validation of IRS-1 transcription (24 h) and protein (48 h) expressions after PCMV7.1-IRS-1 vector transfection in A549 and H1299 cells. (**B**) Proliferation analysis of A549 and H1299 cells after IRS-1 overexpression. (**C**) Colony forming analysis of A549 and H1299 cells after IRS-1 overexpression. (**D**) Wound-healing analysis of A549 and H1299 migration after IRS-1 overexpression. * *p* < 0.05; ** *p* < 0.01.

To further verify the regulatory function of IRS-1 in NSCLC cells, we constructed PCMV7.1-IRS-1 vector (data not shown) and performed IRS-1 overexpression experiments. Here, as IRS-1 overexpression had little effect on LTEP-a2 cell phenotype, the phenotype results were shown only in A549 and H1299 cells. Figure 4A showed the expressions of IRS-1 in A549 and H1299 cells after PCMV7. 1-IRS-1 vector transfection. Compared with control groups, the transcription and protein levels of IRS-1 significantly upregulated after plasmid transfection. Proliferation analysis showed that overexpression of IRS-1 could promote the growth ability of A549 cells, while it had small (but significant) effect on H1299 proliferation (Figure 4B). However, increasing the IRS-1 expression both promoted the colony forming abilities of A549 and H1299 cells, indicating that IRS-1 was more likely to affecting the unanchored growth ability of NSCLC cells (Figure 4C). As expected, overexpression of IRS-1 also significantly accelerated the migration ratio of A549 and H1299 cells (Figure 4D), which was in contrary to the siRNA knockdown results. Notably, although IRS-1 expression is vast at both mRNA and protein levels after vector transfection, the observed effects are rather small at the phenotypic level. This might be due to cell phenotypic variation depending on changes of multiple factors, excessively enhancing the expression of one protein may not have an obvious effect. Meanwhile, we also analyzed the effects of phycocyanin on IRS-1 overexpression A549 and H1299 cells (Figure S2). The results showed that phycocyanin could significantly reduce the proliferation of IRS-1 overexpression A549 and H1299 cells. Taken together, these results showed that overexpression of IRS-1 promotes proliferation and migration of NSCLC cells, suggesting IRS-1 played an important role in phycocyanin-mediated proliferation and migration inhibition process of NSCLC cells.

## 3. Discussion

In this study, we investigated the underlying anticancer mechanism of phycocyanin, a natural pigment mainly exists in Cyanobacteria, Rhodophyta, Cryptophyta, and Glaucophyta [33]. Due to the safety features (no side effects on normal tissue cells) of phycocyanin [19], its antineoplastic ability in lung cancer is of particular interest. A large number of studies have shown that phycocyanin can inhibit the proliferation, cause cell cycle arrest and induce apoptosis of NSCLC cells [34,35]. Previously, our team had discovered that phycocyanin exerted anti-proliferative functions through reducing receptor-interacting serine/threonine-protein kinase 1 (RIPK1) and toll/interleukin 1 receptor domain-containing adaptor (TIRAP), two mediated proteins in multiple NSCLC cells, revealing the potential anticancer mechanism of phycocyanin in NSCLC [36,37]. Here, IRS-1, a key regulatory protein of phycocyanin, was investigated to participate in the growth and migration processes of NSCLC cells.

As a matter of fact, the IRS families have been considered as important signal intermediates in insulin-regulated glucose homeostasis, and play important roles in tissue growth and tumorigenesis [38]. It has been reported that IRS-1 contributes to the excessive growth stimulation in human pancreatic cancer [16]. Moreover, Dearth et al. discovered transgenic mice overexpressing IRS-1 in the mammary gland showed progressive mammary hyperplasia, tumorigenesis, and metastasis [39], which further suggested the oncogenicity of IRS-1. In this work, knockdown of IRS-1 expression resulted in the reduced proliferation and migration of H1299, A549, and LTEP-a2 cells, indicating IRS-1 was involved in regulating the growth of NSCLC cells, which was partially in accord with Dearth’s study. Interestingly, Han et al. reported that 43.6% of NSCLC specimens showed loss of IRS-1 expression, and downregulation of IRS-1 is more frequently observed in squamous cell carcinoma than other type lung cancer [40]. Meanwhile, low IRS-1 cytoplasmic expression also showed a trend toward association with decreased overall survival in squamous cell carcinoma patients [41]. In this study, IRS-1 was relatively highly expressed in A549, H1299, and LTEP-a2 cells, which might be due to these NSCLC cells being adenocarcinoma cells.

The PI3K/AKT signaling pathway is indispensable for cell differentiation, and it has been evolutionarily conserved to maintain appropriate cell growth, survival, and metabolism [42]. IRS-1 is one of the central mediators in transmitting signals from IGF-IR receptors via the PI3K/AKT pathway and it is commonly overexpressed in cancer [43]. Meanwhile, Kathryn E et al. reported that inhibition of mTOR in tumors caused activation of Akt kinase which is associated with induction of IRS-1 and was prevented by IGF-IR inhibition [44]. Strikingly, Gokhan et al. [45] discovered that gain of function mutations of IRS-1 could be related to development of lung cancer, because IRS-1 binds to PI3Kp85 to activate Akt kinase. In the present study, we found that knockdown the expression of IRS-1 could inhibit the activity of PI3K-AKT signaling pathway. Xu et al. showed that PI3K-AKT activity could be inhibited by targeting IRS-1 in colorectal cancer [46], which supported our results. Overall, this work explored the potential anti-growth mechanism of phycocyanin in NSCLC, and discovered that phycocyanin could inhibit the growth and migration, and promote apoptosis of NSCLC cells via downregulating IRS1 expression.

## 4. Materials and Methods

### 4.1. Cell Lines and Culture Condition

Phycocyanin (isolated and purified from Spirulinaplatensis) standard substance was purchased from BinMei Biotechnology (Taizhou, China). Human NSCLC A549, H1299, and LTEP-a-2 cell lines were purchased from American Type Cell Collection (ATCC, Manassas, VA, USA). Cells were maintained at 37 °C in an atmosphere containing 5% CO_2_ in Dulbecco’s modified Eagle medium (DMEM) supplemented by 10% fetal bovine serum (FBS), 0.1 mg/mL of streptomycin, and 100 units/mL of penicillin.

### 4.2. RNA-Seq Analysis

The RNA-seq analysis was performed using HiSeq 4000 (Illumina, San Diego, CA, USA). Briefly, total RNA was extracted using Trizol after phycocyanin treatment (4.8 μM). Five micrograms of total RNA was used for transcriptome analysis. Base calling was adopted to convert original sequencing images to sequential data. The human genome sequence and gene annotations were obtained from the UCSC Genome Website (http://genome.ucsc.edu/, accessed on 14 June 2020). The differentially expressed genes between phycocyanin-treatment and control cells were identified based on fragments per kilobase million reads (FPKM). The control and phycocyanin-treated groups were analyzed in triplicate.

### 4.3. Cell Proliferation Assay

Cell proliferation assay was detected by the MTT method [31]. Briefly, cells (40% cell density) were added into 96-well plate and treated with 4.8 μM phycocyanin for 24 h. 100 μL 10% MTT medium was added to the experimental cells, followed by 100 μL SDS-HCL lysate addition after 4 h at 37 °C. The absorbance value (570 nm) was observed after 15 h of culture.

### 4.4. Cell Colony Formation Assay

Cell colony formation assay was performed according to pervious work [31]. Briefly, cells in the exponential growth phase were harvested and transferred to a six-well plate (20% cell density), followed by 4.8 μM phycocyanin treatment for 10–15 days. The number of cell clones was counted after Giemsa staining.

### 4.5. Cell Cycle Assay

Cells in the exponential growth phase were treated by 4.8 μM phycocyanin for 48 h, then harvested and fixed by 70% cold ethanol for at least 24 h, then resuspended in a 500 µL of propidium iodide (PI)/RNase staining buffer, followed by washing with cold PBS. Cell cycle distribution was measured using FACSCalibur (Becton Dickinson, Franklin Lakes, NJ, USA).

### 4.6. Cell Apoptosis Assay

Cells in the exponential growth phase were treated by 4.8 μM phycocyanin for 48 h, then harvested and resuspended gently in 100 µL binding buffer, followed by adding 5 µL of AnnexinV and 7AAD staining solution, respectively, for 20 min. Non-apoptotic cells cannot be stained by AnnexinV and 7AAD (Annexin V−/7AAD−). Annexin V could bind the cellular phosphotidylserine, which would expose to the surface of cells in early apoptotic process (Annexin V+/7AAD−). 7AAD could bind the nucleic acid of late apoptotic or death cells (Annexin V+/7AAD+). The apoptosis of cells was analyzed by FACS Calibur (Becton Dickinson).

### 4.7. siRNA Transfection

Cells were added into 6-well plate (50% cell density) and cultured at 37 °C for 12 h. 50 nM of siRNAs were transfected into cells according to the manufacturer’s instructions (Dharmacon, Lafayette, CO, USA). Negative siRNA was used as the negative control.

### 4.8. Wound-Healing Assay

Cells in the exponential growth phase were harvested and transferred to 6-well plates until the confluence was 100%. The cell “wound” was scratched by the sterile tips in 6-well plates after 4.8 μM phycocyanin treated. Photos of the wounds were taken every 12 h, and the widths of the wounds were measured at three positions for each replicate using the Leica Application Suite (Leica Microsystems GmbH, Wetzlar, Germany).

### 4.9. Quantitative Real-Time PCR (qRT-PCR) Analysis

Total RNA of cells was extracted with Trizol reagent, followed by reverse transcription with primescript RT master mix. qRT-PCR analysis was performed using Applied Biosystems Step One-Plus (Waltham, MA, USA) and SYBR Green real-time PCR Master Mix Kit (Takara, Dalian, China). GAPDH was used as an endogenous control. The primers were as follows: IRS-1 up 5′-TACTACGAGAACGAGAAG-3′ and down 5′-TTGTTGATGTTGAAGCAG-3′. GAPDH up 5′-AGGGCTGCTTTTAACTCT-3′ and down 5′-TGGGTGGAATCATATTGGA-3′. The relative expression of each gene was calculated and normalized using the 2^−∆∆Ct^ method relative to GAPDH. Each assay was performed in quadruplicate. Three independent experiments were carried out.

### 4.10. Western Blot Analysis

Total protein was extracted from cell by RIPA lysis buffer (1% NP40, 0.1% SDS, 5 mM EDTA, 0.5% sodium deoxycholate, 1 mM sodium orthovanadate) containing 0.1 μM protease and phosphatase inhibitors, and centrifuged at 12,000 r for 45 min at 4 °C. Protein concentrations were measured using the Bradford method. The equivalent amounts of proteins (50 μg) were separated by 12% SDS-PAGE and then transferred to a polyvinylidenedifluoride (PVDF) membrane. Primary antibodies including anti-IRS-1 (1:500), anti-Bax (1:1000), anti-Bcl-2 (1:1000), anti-p-PTEN (1:500), anti-p-PDK1 (1:1000), anti-p-AKT Ser473 (1:500), anti-p-AKT Thr308 (1:1000), anti-C-Raf (1:500), and anti-GSK-3β (1:1000) were incubated with the membrane at 4 °C overnight, followed by washing with PBS for 15 min. Secondary antibody anti-β-actin (1:5000) was then incubated for 30 min at 37 °C and wash the followed by signal detection using an electrochemical luminescence (ECL) system.

### 4.11. Statistical Analysis

The numerical data were expressed as means ± standard deviation (SD). Two-tailed Student’s *t*-test was performed for comparison among the different groups. In addition, *p* < 0.05 (*) or *p* < 0.01 (**) was considered as statistically significant.

## Data Availability

Data sharing is not applicable.

## Supplementary Materials

The following are available online. Table S1: Detailed information of differentially expressed genes (DEGs). Figure S1: Inhibitory effects of phycocyanin (4.8 μM) on A549 cells. Figure S2: Proliferation analysis of A549 and H1299 cells after IRS-1 overexpression and PC treatment.

## Sample Availability

Samples of phycocyanin are available from the authors.
