# Supplementary material for: Insulin Receptor Substrate 1 Is Involved in the Phycocyanin-Mediated Antineoplastic Function of Non-Small Cell Lung Cancer Cells
Source: Molecules. 2021 Aug 4;26(16):4711. doi: 10.3390/molecules26164711 (PMC8401963; doi:10.3390/molecules26164711)
Supplement: Supplementary file 1 [file molecules-26-04711-s001.zip › Supplementary Figure.pdf]

Supplementary Figures

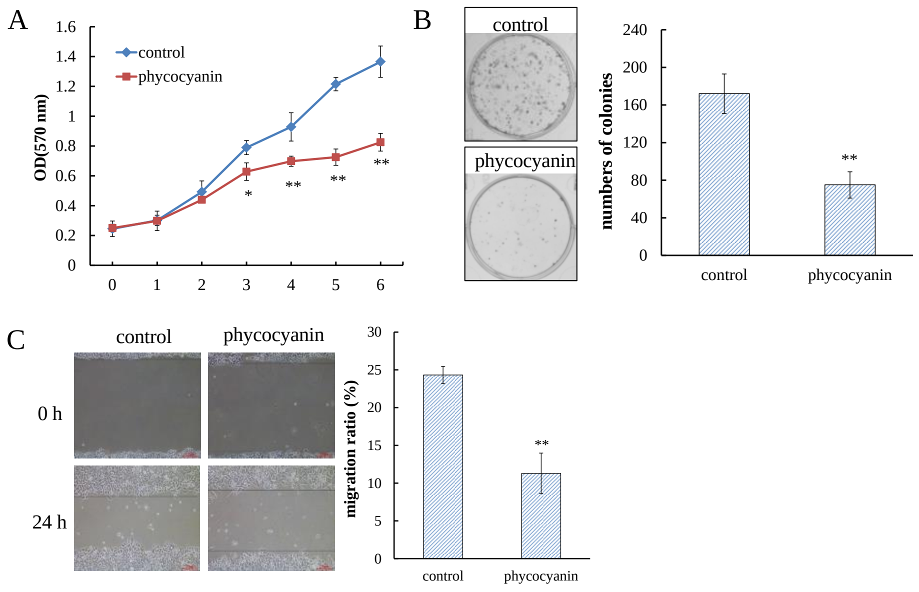

Figure S1. Inhibitory effects of phycocyanin (4.8 μM) on A549 cells. (A) Proliferation analysis of A549 cells after phycocyanin treatment. (B) Cell colony formation analysis of A549 cells after phycocyanin treatment. (C) Cell migration assay of A549 cells after phycocyanin treatment.

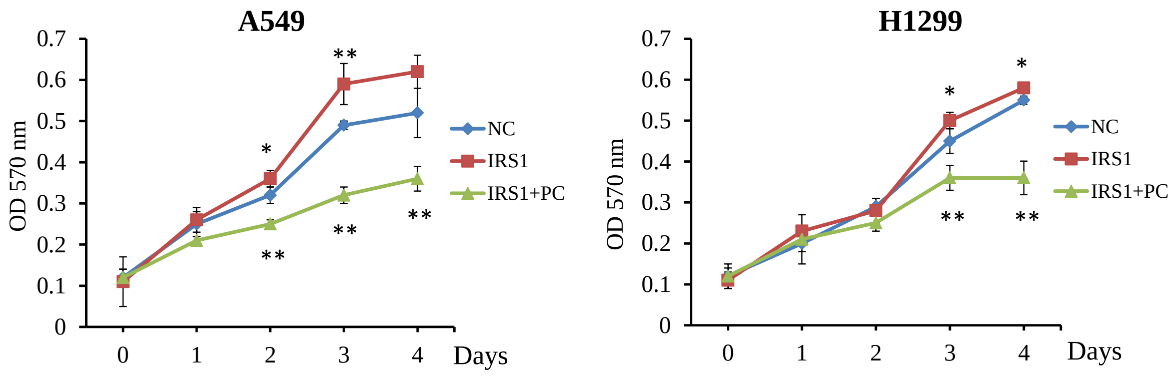

Figure S2. Proliferation analysis of A549 and H1299 cells after IRS-1 overexpression and PC treatment. NC, negative control; IRS1, IRS1 overexpression; IRS1+PC, phycocyanin-treated in IRS1 overexpression cells.
